# *Arabidopsis thaliana cbp80*, *c2h2*, and *flk* Knockout Mutants Accumulate Increased Amounts of Circular RNAs

**DOI:** 10.3390/cells9091937

**Published:** 2020-08-21

**Authors:** Anna Philips, Katarzyna Nowis, Michal Stelmaszczuk, Jan Podkowiński, Luiza Handschuh, Paulina Jackowiak, Marek Figlerowicz

**Affiliations:** 1 Institute of Bioorganic Chemistry, Polish Academy of Sciences, 61-704 Poznan, Poland; aphilips@ibch.poznan.pl (A.P.); kkozlowska@ibch.poznan.pl (K.N.); michal.stelmaszczuk@ibch.poznan.pl (M.S.); jpodkow@man.poznan.pl (J.P.); luizahan@ibch.poznan.pl (L.H.); 2 Institute of Computing Science, Poznan University of Technology, 60-965 Poznan, Poland

**Keywords:** circRNA, *Arabidopsis thaliana*, splicing, *cbp80*, *c2h2*, *flk*, RNA-seq

## Abstract

Circular RNAs (circRNAs) are the products of the non-canonical splicing of pre-mRNAs. In contrast to humans and animals, our knowledge of the biogenesis and function of circRNAs in plants is very scarce. To identify proteins involved in plant circRNA generation, we characterized the transcriptomes of 18 *Arabidopsis thaliana* knockout mutants for genes related to splicing. The vast majority (>90%) of circRNAs were formed in more than one variant; only a small fraction of circRNAs was mutant-specific. Five times more circRNA types were identified in *cbp80* and three times more in *c2h2* mutants than in the wild-type. We also discovered that in *cbp80*, *c2h2* and *flk* mutants, the accumulation of circRNAs was significantly increased. The increased accumulation of circular transcripts was not accompanied by corresponding changes in the accumulation of linear transcripts. Our results indicate that one of the roles of CBP80, C2H2 and FLK in splicing is to ensure the proper order of the exons. In the absence of one of the above-mentioned factors, the process might be altered, leading to the production of circular transcripts. This suggests that the transition toward circRNA production can be triggered by factors sequestering these proteins. Consequently, the expression of linear transcripts might be regulated through circRNA production.

## 1. Introduction

Circular RNAs (circRNAs) are a class of non-coding alternatively spliced RNA transcripts. It has been shown that circRNAs are present across the eukaryotic tree of life [1]. Moreover, many of them are evolutionarily conserved and highly abundant [2,3]. These facts, together with the newest findings on some circRNA functions [4,5,6], strongly suggest important the roles of circRNAs in crucial cellular processes, especially in the regulation of gene expression [7]. In humans and animals, circRNAs may act as protein-binding molecules [7,8,9] or miRNA sponges [6,9,10]. For example, CDR1as/ciRS−7 and *Sry* circRNAs have many miRNA-binding sites, via which they sequester miRNA from its target sites in mRNA. It is also becoming clear that many circRNAs are involved in the pathogenesis of numerous diseases, e.g., cancer [11,12] or Alzheimer’s disease [13]. In addition, several mechanisms of circRNA formation have been postulated. It has been shown that circRNA generation is to some degree dependent on the length and sequence of introns [2,7,14]; however, there are many exceptions to this general rule [1]. It is also known that circRNAs are generated in a cell-specific manner, and their accumulation levels are not correlated with those of corresponding mRNAs [15]. The facts listed above permit us to hypothesize that in addition to the protein/RNA factors involved in canonical splicing, there are other unknown factors that promote circRNA generation. Moreover, as circRNA and mRNA are produced from the same exons by the same machinery (spliceosome), it is highly probable that circRNAs may function in the regulation of gene expression by competing with linear mRNA generation [7]. All the above-mentioned observations come from the study of circRNA in animals and humans. Unfortunately, so far, little is known about the biogenesis and roles of circRNAs in plants. To date, only one plant circRNA has been clearly assigned functions; circRNA derived from exon 6 of the *SEPALLATA3* (*SEP3*) gene was shown to regulate flower development in *Arabidopsis thaliana* [16].

To identify genetic factors that influence circRNA production, we used one of the best-studied model dicot plants, *A. thaliana*. This plant has thousands of easily available mutants, including well-characterized single-gene knockouts. Considering the putative mechanisms of circRNA formation, we selected wild-type *A. thaliana* (Columbia ecotype, abbreviated Col-0) and its 18 variants, each harboring a knockout mutation in a gene that encodes a protein involved in splicing [17]. The compositions of the transcriptomes of all these plants were determined via RNA-seq. Consequently, genes potentially affecting circRNA production were identified.

## 2. Materials and Methods

### 2.1. Plant Material and RNA Extraction

Seeds of Col-0 and T-DNA insertion knockout mutants of *A. thaliana* plants were obtained from Arabidopsis Biological Resource Center (ABRC). All the ABRC IDs (deposition numbers) with the names of knocked-out genes in mutant plants used in the study are listed in Table S1. Formal identification of the plant material used in the study was performed by the J. Ecker or Bernd Weisshaar laboratory and Syngenta (plant material donors) using Illumina sequencing or PCR.

The seeds of the *A. thaliana* plants were sterilized by placing them in filter tubes and washing with 70% ethanol, distilled water, 0.01% Amistar 250 SC (Syngenta, Warsaw, Poland), and again with distilled water using a vacuum pump. Next, the seeds were stratified for 4 days at 4 °C in 0.1% agarose solution. Plants were grown using Arasystem (Betatech, Ghent, Belgium) and Jiffy-7 peat pellets in a growth chamber with 16 h of light at 23 °C and 8 h of dark at 18 °C. Whole leaves at stage 3.90 (rosette growth complete) [18] were collected, frozen in liquid nitrogen, and stored at −80 °C until genotyping and RNA isolation.

Prior to obtaining RNA, mutant plants were genotyped using a strategy proposed by the Salk Institute Genomic Analysis Laboratory for *A. thaliana* T-DNA insertion mutants. Briefly, the T-DNA Primer Design Tool was used to obtain primer sequences for each mutant. All the primers (Genomed, Warsaw, Poland) used in genotyping reactions are listed in Table S1. Next, with a forward border primer (FBP) targeting the T-DNA insertion and forward (FP) and reverse (RP) primers that flanked the T-DNA insertion site, PCRs with genomic DNA (gDNA) from the leaves of mutant and Col-0 plants (as a control) were performed.

The PCR mixtures for the genotyping reaction (25 μL of the total volume) contained 10 ng of gDNA, 0.625 U of Taq DNA polymerase (5 U/µL), 2.5 mM MgCl_2_, 0.2 mM dNTPs, 1× Taq Buffer with (NH_4_)_2_SO_4_ (Thermo Fisher Scientific, Waltham, MA, USA), and each primer at 0.5 µM (Genomed, Warsaw, Poland). PCR was performed in a T-100 thermal cycler (Bio-Rad, Hercules, CA, USA) using the following program: preheating at 95 °C for 3 min, followed by 25 cycles of 95 °C for 30 s, 62 °C for 30 s and 72 °C for 1 min and 15 s. Finally, the reaction mixtures were subjected to elongation at 72 °C for 5 min. The QIAquick PCR Purification Kit (Qiagen, Hilden, Germany) was used for the purification of PCR products, which were then resolved on a 2% agarose gel for 70 min at 120 V in a Wide Mini-Sub Cell GT System (Bio-Rad, Hercules, CA, USA), and subsequently stained with a 0.5 µg/mL EtBr solution for 20 min on a rocker. A ChemiDoc XRS+ System (Bio-Rad, Hercules, CA, USA) was used to obtain gel images, which were analyzed with Image Lab software (Bio-Rad, Hercules, CA, USA).

Total RNA was isolated from 100 mg of powdered leaf samples from 4 biological replicates for each mutant and wild-type plant using the mirVana miRNA Isolation Kit (Thermo Fisher Scientific, Waltham, MA, USA). For each replicate, 10 plants were used to obtain powdered samples. Subsequently, 10 μg of total RNA was treated with 2 U of Turbo DNase (Thermo Fisher Scientific, Waltham, MA, USA) and purified with the QIAquick Nucleotide Removal Kit (Qiagen, Hilden, Germany). Capillary electrophoresis (2100 Bioanalyzer, Agilent, Santa Clara, CA, USA) with the Plant RNA Nano Assay was used to assess RNA quality and integrity. For further analyses, we used samples with an RNA integrity number (RIN) >7.5.

### 2.2. Library Preparation and Sequencing

For library preparation, we used 2.5 μg of total RNA (isolated as described above) treated with the Ribo-Zero rRNA Removal Kit (Plant Leaf) (Illumina, San Diego, CA, USA) following the manufacturer’s recommendation. The level of rRNA depletion was determined by capillary electrophoresis (2100 Bioanalyzer, Agilent, Santa Clara, CA, USA) with the Plant RNA Pico Assay.

One hundred-nanogram aliquots of RNA samples were prepared in 4 biological replicates. Next, libraries were obtained separately using Kapa strand RNA-Seq library synthesis with NEB adapter modification. The libraries were subjected to qualitative analysis using capillary electrophoresis (2100 Bioanalyzer, Agilent, Santa Clara, CA, USA) with the High Sensitivity DNA Assay and quantitative analysis with a Qubit fluorometer (Invitrogen, Carlsbad, CA, USA) prior to sequencing. The samples were sequenced with the Genome Analyzer IIx (Illumina) and 108-bp paired-end protocol.

### 2.3. CircRNA Identification and Quantification

Remnant adapter sequences were removed, low-quality bases at read ends were trimmed out (minimum quality score of 30; 99.9% base call accuracy) and reads shorter than 20 nucleotides were removed with AdapterRemoval (version 1.5.4; [19]).

CircRNAs were identified using a protocol proposed [20]. Briefly, filtered reads were mapped with BWA mem (version 0.7.10; [21]) to the reference genome (TAIR10), and based on back-spliced read identification, CIRI2 generated a list of circRNA candidates supported by at least 2 back-spliced reads. Additionally, the results obtained with CIRI2 were validated with find_circ [9].

Subsequently, the back-spliced reads supporting circRNAs were normalized to the library size of a sample reduced by reads mapped to chloroplasts and rRNA. Reads mapped to rRNA were determined using SortMeRNA (version 2.1; [22]). Plots were generated using the R package (version 3.6.1) with ggplot2 and wiggleplotr libraries.

### 2.4. Transcript-Level Expression and Alternative Splicing Analysis

Filtered reads were mapped to the reference genome (TAIR10) using HISAT2 (version 2.1.0; [23,24,25]). Transcript assembly and quantification was assessed with StringTie (version 1.3.3b [23]). Differential expression analysis of the quantified transcripts was made using the DESeq2 library (version 1.26.0; [26]). Plots were generated using the R package (version 3.6.1) with ggplot2 and wiggleplotr libraries.

## 3. Results

### 3.1. Selection of the A. thaliana Knock-Out Mutants

In this study, we analyzed circRNA and mRNA accumulation in wild-type *A. thaliana* and its selected mutants that had changes in the genes involved in splicing. We chose these mutants as the primary objects of our analyses because the newest findings indicate that transcript circularization is regulated by general splicing factors, cis-elements and cognate factors, but with unknown regulatory rules that are distinct from those of canonical splicing [1]. According to the Arabidopsis Splicing Related Genes (ASRG) database, 395 genes encode splicing-associated proteins in *A. thaliana* [17]. Preliminary *A. thaliana* variant selection led us to a list of 86 mutants with changes in genes related to splicing, of which 23 had a precisely described phenotype (data from the ABRC [27]). Furthermore, we narrowed the set of mutants to those with a Col-0 background so as to obtain a common reference for all of the analyzed plants. We also excluded heterozygotes and variants with lethal mutations. As a result, the studied plants included wild-type *A. thaliana* and its 18 mutants listed in Table S1. All plants were cultivated and genotyped as described in the Materials and Methods.

### 3.2. CircRNA Identification in Wild-Type and Mutant Plants

RNA samples were isolated from the leaves of all selected *A. thaliana* variants, and RNA-seq libraries were prepared using total RNA depleted of rRNA. For each plant, RNA-seq experiments were performed in four biological replicates (Materials and Methods). On average, 60,707,438 paired-end reads were obtained for the library; 99.99% of the reads passed trimming and quality filtration, 95.95% of them mapped to the *A. thaliana* reference genome (TAIR10), and 7.81% of the reads mapped to the rRNA sequences (Table S2). This observation was consistent with the capillary electrophoresis results (Figure S1).

For circRNA identification, we used the protocol proposed by Gao et al. (CIRI2 [20]) as the best-performing de novo identifying program [28,29]. In total, we identified 30,923 unique circRNAs in Col-0 and all mutants. For each *A. thaliana* variant, we observed that a large fraction of circRNAs (from 84.54% in *cbp80* up to 90.72% in the *tri-20* mutant) was generated in only one biological replicate. CircRNAs that were identified in all four biological replicates were rare (from 0.91% in *tri-20* to 2.62% in *cbp80*) (Figure S2; Table S3A).

The results generated by CIRI2 were additionally validated with find_circ, one of the most popular programs for circRNA identification [9]. The outcomes of both analyses were consistent (Figure S2A,B and Table S3B), and with find_circ most of the circRNAs were detected in one biological replicate (75.70%) while only a small fraction of the circRNAs was identified in all four replicates (9.60%). In total, 82% of the circRNAs detected by CIRI2 were also found by find_circ. Of note, find_circ generally identified 1.6 times more circRNAs. This discrepancy might be a result of the different circRNAs identification strategies employed by each program [29], and had no influence on the general conclusion that the majority of circRNAs produced in *A. thaliana* are generated stochastically. To exclude the possibility that the observed phenomenon was caused by the low quality of next-generation sequencing (NGS) data, we checked whether the mRNA accumulation in biological replicates was also variable (see Table S3C,D). In contrast to circRNA, the Pearson correlation coefficients calculated for mRNA were very high, on average r = 0.9956 (0.9738–0.9999), while the Pearson correlation coefficients calculated for circRNAs were significantly lower, on average r = 0.5454 (from 0.1321 to 0.9773). Thus, the results of the above described mRNA analysis proved that the quality of the NGS data used in our studies was very high. These results are consistent with the observation made in our previous study [30] that most circRNAs in *A. thaliana* are generated stochastically, and thus probably have no biological function. This is why we decided to further focus only on reproducibly generated circRNAs that were present in all four biological replicates.

**Figure 1:**
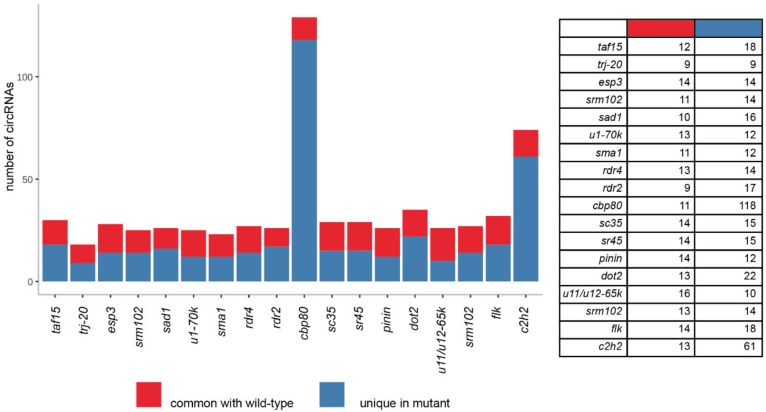
The numbers of circRNAs identified in *A. thaliana* mutants in 4 biological replicates. CircRNAs unique to Col-0 were not shown.

**Figure 2:**
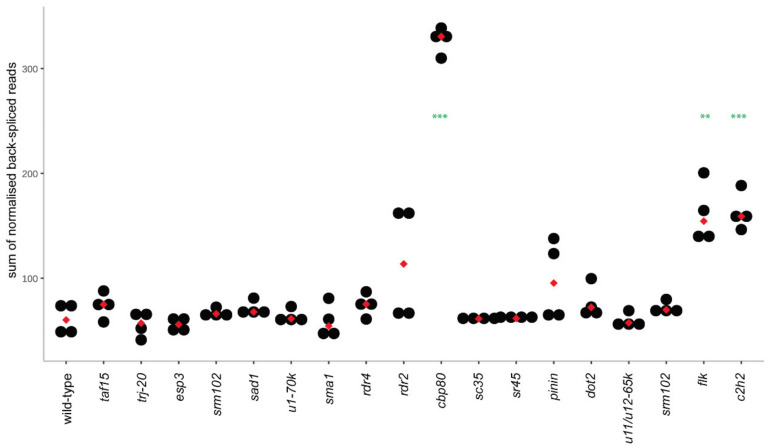
Level of circRNA accumulation in the wild-type and mutant plants [4 biological replicates, **p-value ≤ 0.01, ***p-value ≤ 0.001).

In total, 24 different circRNAs were identified in the leaves of the wild-type plant. Interestingly, three of these circRNAs (AT1G12240, AT2G44920, AT2G14050) were unique and not present in any other studied variant. All 24 circRNAs were previously reported in PlantcircBase [31], and 16 of them were reported in AtCircDB [15]. Moreover, to validate these results, we checked if the identified circRNAs were also found in a similar analysis that we performed earlier for RNA samples isolated from Col-0 and treated with RNase R prior to library preparation [30]. In total, 22 of them were identified in those libraries. On average, 35 circRNAs were identified per mutant plant (Figure S1). However, we observed that the *cbp80* mutant had a very high number of identified circRNAs (129, Figure 1). The *c2h2* mutant also had a significantly higher-than-average number of circRNAs identified (74). In terms of circRNA abundance, three mutants, namely *cbp80*, *flk* and *c2h2*, accumulated significantly more circRNAs than the wild-type and remaining mutants (Figure 2). The tendency was upheld for *cbp80* and *c2h2* variants when considering all circRNAs produced by the mutants, including the sporadic circRNAs identified in only one biological replicate (Figure S3). This observation suggests that the mutations affect the total back-splicing process. Notably, all identified circRNAs had canonical splicing signals at the back-splice site (CIRI2 requires the presence of a canonical splicing site to call a circRNA).

### 3.3. CircRNAs in the cbp80 Mutant

The landscape of circRNAs in the *cbp80* mutant is much more complex than that in the wild-type plant. Among the 129 detected circRNAs, only 11 were common to Col-0, and 26 had at least one mutant (Figure 1, Figure S4). A total of 118 circRNAs seem to be typical to only this mutant, compared with the wild-type. Only 43.2% (51) and 19.5% (23) were previously reported in databases (*PlantcircBase* and *AtCircDB*, respectively).

**Figure 3:**
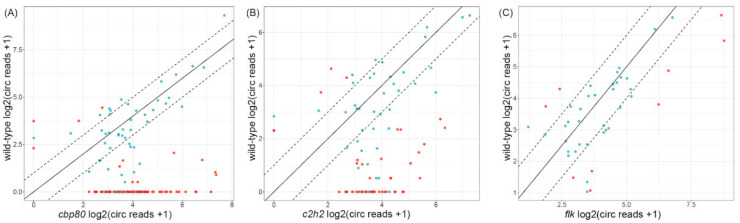
Pairwise comparison of circRNA accumulation in the wild-type plant and (**A**) *cbp80*, (**B**) *c2h2* and (**C**) *flk* mutants. Dashed line: 2-fold cut-off; circRNAs with *p*-value ≤ 0.05 are marked in red.

First, to validate the above results, we verified which of the 118 circRNAs were exclusively specific to *cbp80*. Alternatively, the circRNAs could have been present in Col-0 but in very low amounts, thereby not passing the threshold, as they were not identified in all 4 biological replicates. In fact, 87 circRNAs present in the *cbp80* mutant were unique, and were detected in none of the Col-0 biological replicates. Despite the criteria used, gene ontology (GO) enrichment analyses revealed that genes producing *cbp80*-specific circRNAs were enriched in response to stress (response to cold temperature; *p*-value < 0.001; Table S4A,B). The remaining 53 circRNAs were common to Col-0. Among these circRNAs, the accumulation of two circRNAs was reduced in the *cbp80* mutant, and that of 8 was increased (Figure 3A; *p*-value ≤ 0.05 and equal to or greater than a two-fold change).

**Figure 4:**
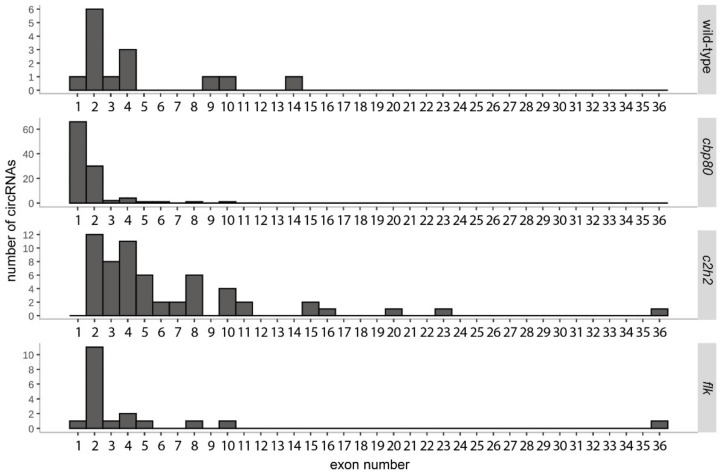
Distribution of exons in which circRNAs start in the wild-type plants and *cbp80*, *c2h2*, and *flk* mutants.

We also examined the exon composition of circRNAs from Col-0 and the *cbp80* mutant, and we noticed that most circRNAs identified in Col-0 started from the second or consecutive exons, and those in the *cbp80* mutant started from the first exon (Figure 4). Similar observations were made when the criterion for circRNA identification was relaxed to include circRNAs present in at least 3 biological replicates (see Figure S5). Interestingly, none of the circRNAs identified in the *cbp80* mutant that included the first exon were found in the other 2 mutants that showed increased circRNA accumulation in comparison with the wild-type plant. Moreover, the structure of circRNAs present in the wild-type (Figure S6A) differed significantly from the one observed for circRNAs unique to *cbp80* (Figure S6B). In total 84.5% (109) of *cbp80* circRNAs started or ended in a known exon, whereas in Col-0 56% (14). This observation suggests that alternative splice sites were preferred in the mutant.

**Figure 5:**
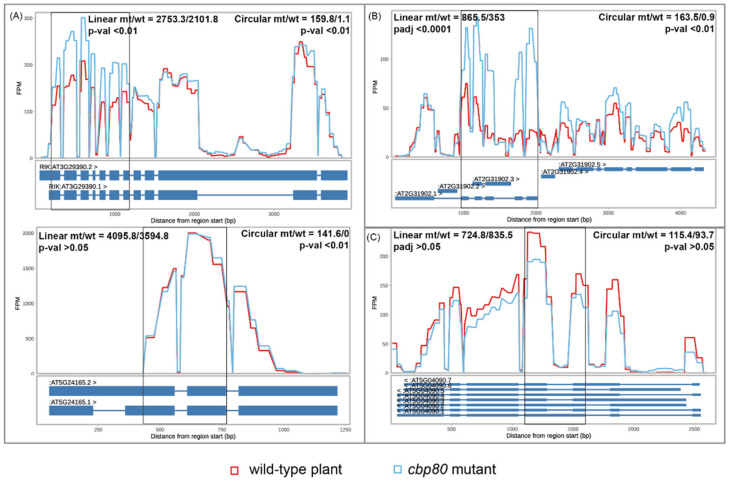
Coverage of genes* giving rise to circRNAs when (**A**) accumulation of circRNA was increased but parent gene expression was not changed (AT3G29390, 3:11289661-11291634—upper panel; AT5G24165, 5:8188571-8188956—bottom panel), (**B**) accumulations of circRNA and parent gene expression were increased (AT2G31902, 2:13561602-13563811), and (**C**) accumulations of circRNA and parent gene expression were not changed (AT5G04090, 5:1106879-1107381). CircRNA ranges are shown in black rectangles. The change of linear RNA accumulation is expressed by the fold change and *p*-value in the left-top of each chart. The change in the circRNA accumulation level is shown by the fold change and *p*-value in the right-top of each chart. * the reads corresponding to both linear and circular RNAs were taken into account.

Finally, we studied the gene expression profile in the *cbp80* mutant and found that the global gene transcript abundance was not increased compared with that observed for Col-0 (Figure S7). Only 8 genes giving rise to circRNAs in this mutant were downregulated, and 16 were upregulated (Table S5A). No statistically significant GO terms were identified for those differentially expressed genes. Last, we checked the global abundance of the linear counterparts of circRNAs in the *cbp80* mutant, and we observed a statistically significant increase in their abundance (*p*-value ≤ 0.05, Figure S8). However, the Pearson correlation coefficient between circular and linear transcripts was 0.015504, indicating that the production of circRNAs was independent of the production of their linear counterparts. The above-mentioned results are clearly reflected in the gene coverage of reads mapped to these genes. There are significant differences in the mapped read number for exons that span circRNAs with increased abundance in the mutant. In Figure 5, we show six examples of genes producing circRNAs with the following features: (i) gene expression level was not changed, but the production of circRNA was significantly increased in the *cbp80* mutant (Figure 5A); (ii) gene expression and circRNA accumulation level was significantly increased in the *cbp80* mutant (Figure 5B); (iii) gene expression and circRNA accumulation level was not changed (Figure 5C). Of note, two of them (namely 3:11289661-11291634 and 5:8188571-8188956) were examples of non-canonical back-splicing occurring in the middle of known exons/introns.

### 3.4. CircRNAs in the c2h2 Mutant

We identified 74 circRNAs in the *c2h2* mutant, which is three times more than the number of circRNAs in the wild-type plant. Among these circRNAs, 13 were common to Col-0 (Figure 1). Sixty-one circRNAs seem to be typical to only this mutant, compared with the wild-type (Figure S4). GO analyses revealed that the genes giving rise to these unique circRNAs were significantly enriched in protein transport and localization (*p*-value < 0.05, Table S4C). Totals of 80.3% (49) and 54.1% (33) of the circRNAs were previously reported in PlantcircBase and AtCircDB, respectively.

Again, to validate the results, we checked whether the unique circRNAs were present in Col-0 but in very low amounts, and thus might not have passed the threshold for being identified in all four biological replicates. Twenty-eight circRNAs present in the *c2h2* mutant were unique and detected in none of the Col-0 biological replicates. GO analyses revealed that genes giving rise to these unique circRNAs were significantly enriched in cellular processes (*p*-value < 0.0001; Table S4D). Among the 55 circRNAs found in both the mutant and Col-0, 3 showed decreased accumulation in the mutant and 15 showed increased accumulation (Figure 3B; *p*-value ≤ 0.05 and greater than or equal to a two-fold change). No statistically significant enrichment of genes giving rise to these circRNAs was observed in GO analyses.

CircRNAs from the *c2h2* mutant most frequently started in the second or later exon, as was observed for Col-0, regardless of the circRNA identification criterion used (circRNAs identified in four of the four biological replicates, or in three of four). (Figure 4, see Figure S5). Moreover, the majority of these circRNAs (62, 83.8%) started or terminated within a known exon/intron (Figure S6C).

The global abundance of transcripts in the *c2h2* mutant was not increased (Figure S7). The expressions of only three genes producing circRNAs were downregulated (AT1G60590, AT3G46970, AT2G21320; Table S5B). Again, we observed no correlation between circular and linear transcript production (Pearson correlation coefficient of −0.075489266). Moreover, the abundance of the linear counterparts of the circRNAs was increased in the *c2h2* mutant (*p*-value ≤ 0.05, Figure S8).

### 3.5. CircRNAs in the flk Mutant

In total, 32 circRNAs were identified in the *flk* mutant. All of these circRNAs were also present in Col-0; however, 18 circRNAs did not pass the criterion of being identified in all 4 biological replicates of Col-0 (Figure 1). Among the *flk* circRNAs, 94.4% (17) and 66.7% (12) were previously reported in PlantcircBase and AtCircDB, respectively. In contrast to the *cbp80* and *c2h2* mutants, no circRNAs specific to this mutant were identified (Figure S4). The analyses of circRNA abundance revealed that the accumulation of seven circRNAs was increased, and that of two was decreased in comparison to their abundance in Col-0 (Figure 3C; *p*-value ≤ 0.05 and a greater than two-fold change). No statistically significant GO enrichment was observed.

Most circRNAs in the *flk* mutant started in the second exon, as in Col-0 (Figure 4). Similar observations were made when the criterion for circRNA identification was relaxed to include circRNAs present in at least three biological replicates (see Figure S5). Similarly to Col-0, 62.5% (20) of the circRNAs started or ended within a known exon/intron (Figure S6D).

The general abundance of transcripts in the *flk* mutant was not increased (Figure S7), and no correlation between circular and linear counterparts was observed (Pearson correlation coefficient of −0.05346). Only one gene (AT4G30975) giving rise to circRNAs in the *flk* mutant was upregulated (Table S5C). On average, the abundance of the linear counterparts of circular transcripts was increased (*p*-value ≤ 0.001, Figure S8).

### 3.6. CircRNA Production as a Form of Alternative Splicing

Considering that splicing machinery is involved in circRNAs biogenesis, we checked whether the production of alternatively spliced linear transcripts was also altered in the analyzed *A. thaliana* variants. The analysis of all splicing variants identified in the studied plants revealed that on average, 8.9% of the transcripts (standard deviation: st-dev. 3.7%) per mutant displayed significant differences in the levels of expression compared to the wild-type (Figure S9). Mutant *u11/u12-65k* was characterized by a very low number of alternatively spliced transcripts (as well as a very low number of circRNAs, specifically, 10 if circRNAs identified in four biological replicates in the wild-type were considered and 0 if all circRNAs in the wild-type were considered), suggesting that the mutation did not significantly affect splicing. The three mutants *rdr4*, *cbp80* and *c2h2* had respectively 1.6-, 2.2- and 1.7-fold more alternatively spliced transcripts than the average value determined for all 18 mutants. Interestingly, we identified *cbp80* and *c2h2* mutants as producing higher amounts and numbers of circRNAs in comparison to Col-0 (Figure 1).

We analyzed the gene structure of the top 10 alternatively spliced transcripts, and noticed that mutant plants generated forms of transcripts other than Col-0 for these genes (Figure S10A,B). When only genes producing circRNAs (identified in all four biological replicates) were considered, we again noticed significant alternative splicing events in the *cbp80* and *c2h2* variants (Figure S11). In *cbp80*, 38 transcripts were identified as up- (20) or down- (18) regulated, and in *c2h2*, 3 transcripts were significantly upregulated and 6 downregulated. Moreover, in these two mutants, in contrast to other analyzed variants, we found transcripts (21 in *cbp80* and 8 in *c2h2*) the level of which displayed small (less than two-fold) but statistically significant changes in comparison to the wild-type plant (padj ≤ 0.05, Figure S11). We checked whether there were exon skipping events that could be coupled to back-splicing (where the skipped exon forms a circRNA), however we found no evidence of such a phenomenon.

## 4. Discussion

CircRNAs have been identified in multiple plant species under stress conditions (reviewed in [32]). Several studies have provided evidence that circRNA pools are dynamic and fluctuate in response to external stimuli. Based on these observations, circRNAs have been proposed to possess biological functions [5,6,7]. Although very intriguing, until now, such hypotheses have been supported by only one direct experimental proof of the functional relevance of circRNA in plants [16]. Considering that circRNAs do play important roles in animal and human cells, evidence of their functions in plants is expected.

CircRNA biogenesis in plants is associated with splicing; however, the molecular mechanism of circRNA formation has not been fully elucidated thus far. To better understand this phenomenon, we analyzed 18 *A. thaliana* knockout mutants for genes that encode proteins involved in different stages of splicing. We demonstrated previously [30], as well as in this study, that only a very limited fraction of circRNAs (less than 3%) are generated in a reproducible manner, and thus can serve as functional molecules. Consequently, the majority of circRNAs most likely reflect the imperfections of splicing. This observation raises questions about the mechanisms that prevent circRNA interference with RNA metabolism, RNA–protein interactions, and RNA-dependent regulatory pathways. Further studies of circRNA intracellular localization are required in order to track the fates of circRNAs and dissect the appropriate surveillance systems.

The production of circRNAs in three mutants was significantly different from that observed in the wild-type and other variants. The first mutant lacked the functional *cbp80* gene, the product of which is a component of the cap-binding complex. The second mutant, *c2h2*, lacked a protein of the U4/U6.U5 tri-snRNP pre-assembled spliceosomal complex that plays a key role in the formation of a catalytically active spliceosome. The third mutant, *flk*, lacked a member of the group of nuclear ribonucleoproteins (hnRNPs), which, if bound to pre-mRNA molecules, serve as a signal that the pre-mRNA is not yet fully processed, and therefore not ready for export to the cytoplasm. The common feature of these three mutants was the increased accumulation of circular transcripts (compared to that in the wild-type plant), which was not accompanied by corresponding changes in gene expression. The number of alternative splicing events was increased in these mutants. The observed higher accumulation of circRNA could be due to either more frequent back-splicing or increased circRNA stability in mutants. This problem requires further detailed studies, and one has to acknowledge that both mechanisms can shape the circRNA pool. However, the fact that we did detect increased numbers of alternatively spliced linear transcripts in mutants indicates that the splicing itself was affected by the gene knockout (currently there is no evidence that these linear alternatively spliced variants could display higher stability than their canonically spliced counterparts). Altogether, these findings suggest that the expression of linear transcripts might be regulated through circRNA production. In the majority of the analyzed mutants, the depletion of splicing-related proteins did not have a significant effect on the generation of circRNAs. This observation indicates that there exist only defined checkpoints prone to either disturbance or regulation, which can change the balance between linear and circular transcripts.

Among the 18 plant variants tested in this study, *cbp80* was found to produce the largest number of unique circRNAs. CBP80 together with CBP20 forms a cap-binding complex (CBC), implicated in splicing and miRNA biogenesis. *A. thaliana* mutants lacking CBC or its components accumulate partially spliced transcripts [33] and pri-miRNA [34]. In addition, the lack of CBC and, in particular, CBP80 affects alternative splicing, especially the selection of the 5′ splice site of the first intron [35]. Our data provide, for the first time, evidence that the absence of CBP80 not only disturbs the production of linear transcript isoforms, but also dramatically increases the generation of circRNAs. Notably, the majority of *cbp80*-specific circRNAs included the first exon, while those formed in other mutants and Col-0 were produced mostly from the second or consecutive exons. This observation is consistent with previous findings that CBP80 preferentially exerts its effect on splicing in the mRNA region proximal to the 5′ end.

CBP80 is involved in abscisic acid (ABA) signal transduction [36] and the flowering pathway [37,38]. Mutants lacking CBP80 display an ABA-induced elevation of cytosolic calcium levels in guard cells, leading to enhanced stomatal closure and the provision of increased drought tolerance [36]. These plants also show an early-flowering phenotype, resulting from the defective splicing of FLOWERING LOCUS C (FLC) introns, which decreases the level of properly spliced FLC transcripts [37]. We wondered whether the generation of circRNAs in *cbp80* also contributed to the observed phenotypes; however, our preliminary analyses did not provide any clues. The repertoire of *cbp80*-specific circRNAs has to be examined in greater detail in order to provide definitive conclusions regarding this aspect.

CircRNAs can either be generated in a controlled way or constitute products of mis-splicing. Based on our data, one can hypothesize that, in general, the undisturbed functioning of protein factors involved in splicing favors the formation of linear transcripts. Such a model appears consistent with the cellular RNA metabolism pathways, which to a large extent rely upon exonucleases, implicated both in quality control processes and in the regulation of transcript stability. Nevertheless, our results suggest that a remodeling of the splicing-related protein complexes may provide a facile mechanism to trigger a switch toward circRNA production. The dynamic composition of the cap-binding protein complex in *A. thaliana* is well documented, and changes during the growth cycle [39]. CBP80 is among the proteins that remain associated with the mRNA cap in both proliferating and quiescent cells, but it changes subcellular localization. During the early stages of the growth cycle, this protein preferentially localizes to the cytoplasm, while later, it accumulates in the nucleus. In turn, its partner, CBP20, first localizes to both the nucleus and the cytoplasm, and then is concentrated in the nucleus [39]. One can hypothesize that some splicing events early in the growth cycle may occur under CBP80 deficiency. Such a situation, according to our results, may activate the formation of circRNAs. Therefore, the recruitment of diverse splicing-related factors (for example, factors dependent on the strength of splicing signals or enhancers) or the sequestration of the components of splicing machinery emerge as potential mechanisms of gene expression regulation in plants. Interestingly, the previous observation that circRNA expression increases when core spliceosomal components are depleted in *Drosophila* [40] suggests the conservation of such a mechanism across kingdoms. Detailed studies are required to provide further insight into this issue in *A. thaliana*.

## Supplementary Materials

The following are available online at https://www.mdpi.com/2073-4409/9/9/1937/s1, Figure S1: The efficiency of Ribo-Zero treatment, Figure S2: Number of identified circRNAs by (A) CIRI2, and (B) find_circ in 1,2,3, or 4 biological replicates, Figure S3: Level of circRNA accumulation in the wild-type and mutant plants, Figure S4: Number of common and unique circRNAs identified in the wild-type and mutant plants, Figure S5: Number of circRNAs, identified in minimum 3 biological replicates, starting with the specified exon number in the wild-type plant, *cbp80*, *c2h2*, and *flk* mutants, Figure S6: The occurrence of canonical and non-canonical back-splicing sites in the wild-type, *cbp80*, *c2h2*,and *flk* mutants, Figure S7: Level of gene expression in the wild-type plant and *cbp80*, *c2h2*, *flk* mutants, Figure S8: Accumulation level of circRNAs’ linear counterparts in the wild-type and mutant plants, Figure S9: The percentage of differentially expressed transcripts in mutants compared to the wild-type plant, Figure S10: The percentage of differentially expressed transcripts in mutants compared to the wild-type plant, Figure S11: The expression profile of transcripts from genes generating circRNAs (identified in 4 biological replicates) in mutants vs. wild-type plants. Table S1: List of primers used in the study, Table S2: The information for the RNA-seq libraries, Table S3: A) CircRNAs identified with CIRI2; B) CircRNAs identified with find_circ; C) Number of normalized reads for linear transcripts; D) Pearson correlation coefficient of mRNA reads between biological replicates, Table S4: Gene ontology for genes giving rise to circRNA, Table S5: A) List of circRNA identified in *cbp80* in 4 biological replicates; B) List of circRNA identified in *c2h2* in 4 biological replicates; C) List of circRNA identified in *flk* in 4 biological replicates. The datasets supporting the conclusions of this article are available in the National Center for Biotechnology Information Sequence Read Archive repository.
