# Supplementary material for: Arabidopsis thaliana cbp80, c2h2, and flk Knockout Mutants Accumulate Increased Amounts of Circular RNAs
Source: Cells. 2020 Aug 21;9(9):1937. doi: 10.3390/cells9091937 (PMC7564263; doi:10.3390/cells9091937)
Supplement: Supplementary file 1 [file cells-09-01937-s001.zip › SM/Supplementary figure 1.pdf]

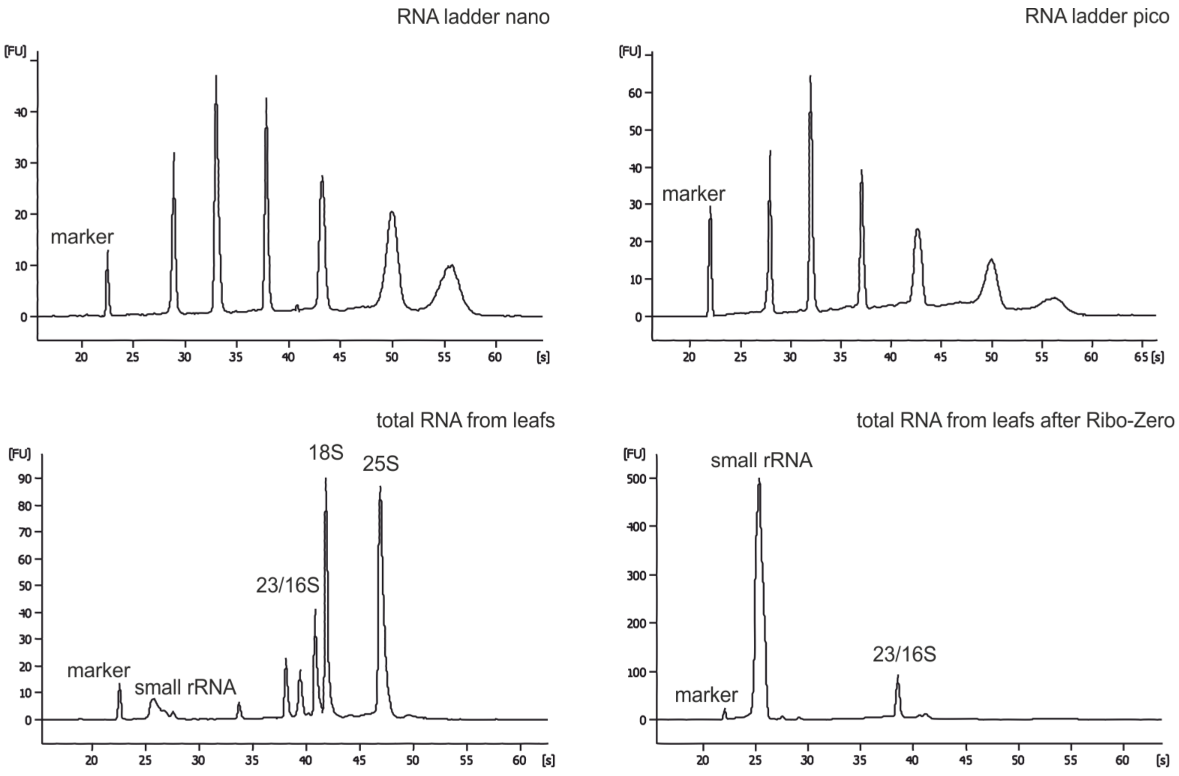

Supplementary Figure 1.
The efficiency of Ribo-Zero treatment.

Representative electropherograms (Bioanalyzer, Agilent) of total RNAs (left side), total RNAs after Ribo-Zero treatment (right side) from leaves of *A. thaliana*. One hundred nanograms of DNase-treated total RNA of each sample was analyzed on the Bioanalyzer, Agilent with RNA Nano Chip in accordance with the manufacturer's instructions. After Ribo-Zero treatment, 3 ng of each sample was analyzed on the Bioanalyzer, Agilent with RNA Pico Chip in accordance with the manufacturer's instructions. The x-axis represents the resolving time in seconds (s), and the y-axis represents fluorescence (fu). In addition to the cytoplasmic 25S and 18S rRNA peaks, other peaks corresponding to 23S and 16S rRNA from chloroplasts and small rRNAs are present.
