# Supplementary material for: Arabidopsis thaliana cbp80, c2h2, and flk Knockout Mutants Accumulate Increased Amounts of Circular RNAs
Source: Cells. 2020 Aug 21;9(9):1937. doi: 10.3390/cells9091937 (PMC7564263; doi:10.3390/cells9091937)
Supplement: Supplementary file 1 [file cells-09-01937-s001.zip › SM/Supplementary figure 10.pdf]

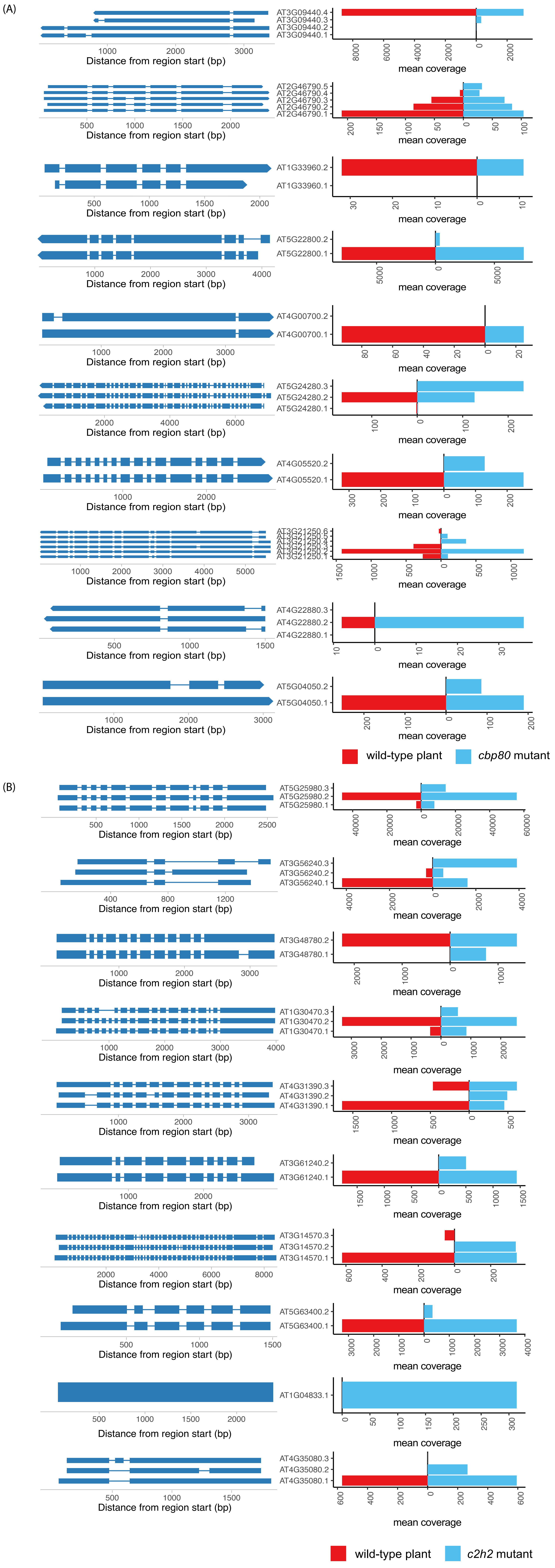

Supplementary Figure 10.

Top 10 differentialy expressed transcripts for (A) *cbp80* and (B) *c2h2* mutants and their gene structure.
