# Supplementary material for: Arabidopsis thaliana cbp80, c2h2, and flk Knockout Mutants Accumulate Increased Amounts of Circular RNAs
Source: Cells. 2020 Aug 21;9(9):1937. doi: 10.3390/cells9091937 (PMC7564263; doi:10.3390/cells9091937)
Supplement: Supplementary file 1 [file cells-09-01937-s001.zip › SM/Supplementary figure 3.pdf]

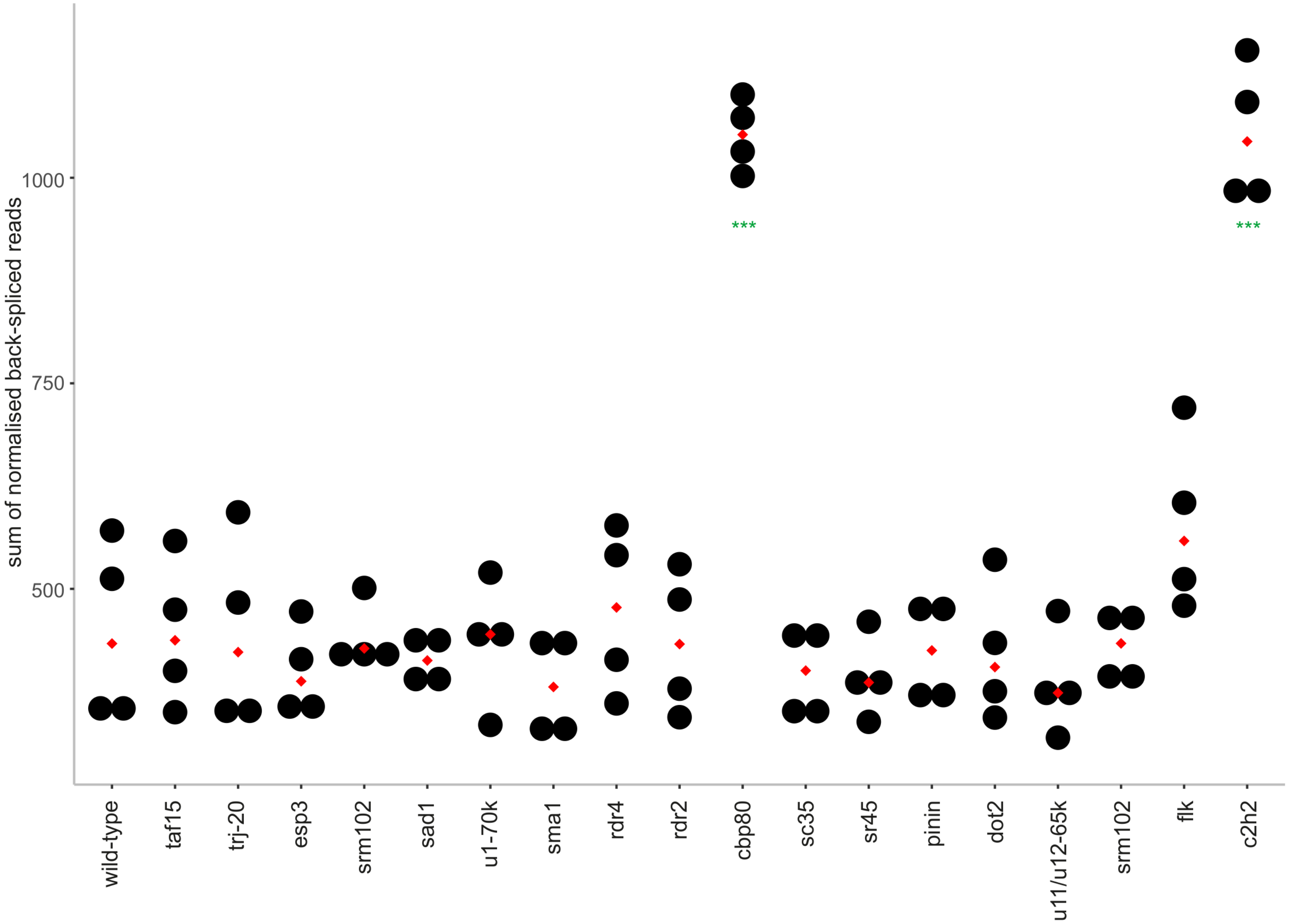


Supplementary Figure 3.

Level of circRNA accumulation in the wild-type and mutant plants (all identified circRNAs, p-value ≤ 0.05 *, ≤ 0.01 **, ≤ 0.001 ***).
